# Should the BCRA1/2-mutations healthy carriers be valid candidates for hematopoietic stem cell donation?

**DOI:** 10.1186/s13053-021-00179-w

**Published:** 2021-04-01

**Authors:** Alberto Fresa, Simona Sica

**Affiliations:** 1 Sezione di Ematologia, Dipartimento di Scienze Radiologiche ed Ematologiche, Università Cattolica del Sacro Cuore, Roma, Largo A. Gemelli 8, 00168 Rome, Italy; 2 Dipartimento di Diagnostica per Immagini, Radioterapia Oncologica ed Ematologia, Fondazione Policlinico A. Gemelli IRCCS, Roma, Rome, Italy

**Keywords:** BRCA, Hematopoietic stem cell, Hematological malignancies

## Abstract

It’s still not clear whether the mutational status of BRCA-mutated healthy hematopoietic stem cells (HSCs) donors could have an impact on the engraftment. Comparing the studies present in literature, we focused on the correlation between BRCA mutations and the development of hematological malignancies and Fanconi anemia (FA); then, we explored HSCs types, frequencies, and functions in the presence of BRCA mutations, as well as the reconstitution of hematopoiesis after chemotherapy and radiation treatments. The role of BRCA mutations in the FA showed a possible involvement in the onset of the disease; the mutation carriers, indeed, did not show any sign of the typical phenotype of the FA. BRCA mutational status can be considered as a risk factor for hematological malignancies, but only for secondary malignancies and/or in the presence of bone marrow stress factors. Currently we don’t know if a conditioning regimen could be compensated by BRCA mutated HSCs, even if murine models tried to show the possible differences between fully mutated, haploinsufficient and normal HSCs. Thus, given the downregulating effect of the mutations on hematopoiesis, it could be questionable to use the HSCs of a BRCA-mutated donor in the presence of another available donor with the same compatibility.

## Background

In 1994 was discovered the first gene associated with hereditary breast cancer, BReast CAncer gene 1 (BRCA1), followed shortly after by BReast CAncer gene 2 (BRCA2). Since then, many of their cellular interactions have been elucidated. They are tumor suppressor genes involved in the DNA double-strand break (DSB) repair mechanism, the mutant phenotypes of which are associated with cancer susceptibility, mostly breast and ovarian cancer [1]. Over the last 25 years, much has been learned about the structure and functions of BRCA gene products. BRCA proteins interact with different regulatory proteins, contributing to the maintenance of genomic integrity. BRCA1 has a central role in forming protein complexes that are required for the repair of DSB and stalled replication forks. BRCA2, interacting with RAD51, is fundamental for the maintenance of cell division and chromosome structure [2–4]. The important role of BRCA genes in hematopoiesis is supported by strong evidence, and, even if much more is known about their involvement, their specific role in hematopoiesis remains uncertain.

In our clinical practice we found ourselves facing a dilemma: should the BCRA1/2 mutated subjects be valid candidates for hematopoietic stem cell (HSC) donation?

This dilemma originated from three consecutive cases of young women BRCA1/2 haploinsufficient, two with acute myeloid leukemia therapy-related and one with Ph + acute lymphoblastic leukemia (who underwent prophylactic bilateral mastectomy at the time of diagnosis), all candidates for allogeneic transplantation. All the possible suitable family donors in these three patients, either HLA identical or haploidentical, happened to be BRCA1/2-mutations healthy carriers, the question of whether it was safe to give their HSC to conditioned patients has become a valuable topic of discussion to talk about.

Comparing data from the studies already present in literature regarding the involvement of BRCA mutations in hematopoiesis, the discussion developed in two different directions. On the one hand, the incidence of hematological malignancies in BRCA1-mutant patients has been investigated, and the role of BRCA mutations in the development of a specific Fanconi Anemia subtype. On the other hand, authors have been studying HSCs types, frequencies, and functions in the presence of BRCA mutations, as well as the reconstitution of hematopoiesis after chemotherapy treatments and in irradiated recipients. We thus examined the available literature to try to find an answer to our question.

## Methods

Many PubMed searches were conducted. To search for possible connections between BRCA mutations and HSC donation, keywords inherent with transplantation and hematopoiesis were entered so the search read “BRCA1 and hematopoiesis” and then “BRCA2 and hematopoiesis.”

Initially, keywords included were: stem cell donation/donor, bone marrow transplantation, allogeneic transplantation, hematopoiesis, hematopoietic reconstitution, hematological malignancies; then all possible variants were entered. Additional searches were conducted for the BRCA mechanism, prevalence, and incidence of cancers. Reference lists of included articles were also manually searched for additional relevant publications.

### Results: Hematological malignancies and fanconi anemia

Fanconi anemia (FA) is a rare autosomal- or X-linked-recessive disease characterized by progressive bone marrow failure, congenital abnormalities, cancer susceptibility and hypersensitivity to DNA cross-linking agents. Its development is associated with specific genes, the coded proteins of which are responsible for the cellular response to DNA damage, along with BRCA1 and BRCA2, thus regulating the DNA repair process. Knockdown of BRCA1 in tumor cells results in an increment of the activation of DNA repair foci, suggesting that BRCA1 is an amplifier of the FA/BRCA pathway. A protein involved in the activation, FANCD2, furthermore interacts with a protein complex in the repair of DNA crosslinks, which is defective in cells from FA patients [5, 6]. The DNA crosslink repair requires S-phase arrest and homologous recombination, and defects of the second one for DNA repair have been found in both BRCA1- and BRCA2-mutant cells [7].

Although BRCA genes involvement in the pathway associated with the development of FA has been extensively described through the years, rarely have been reported cases in which the biallelic mutations of BRCA led to the development of Fanconi anemia. In 2013 and then in 2015 were described two cases in which the biallelic BRCA1 mutations caused a Fanconi anemia-like clinical phenotype, with short stature, microcephaly, and developmental delays [8, 9]. In the anamnesis of both patients there was a solid tumor, either breast or ovarian, and in the second case the chromosomal breakage test was performed with a diagnostic result. Unfortunately, in the first case diepoxybutane test was not available, but significant toxicity to carboplatin was suggestive of hypersensitivity to interstrand crosslink reagents. Even though the two patients didn’t develop bone marrow failure, the authors suggested prudentially to follow hematologic indices in this specific subset of patients. Besides, after the report of the first case, D’Andrea A.D. invited clinicians to test for BRCA1/2 mutation any woman with a history of breast or ovarian cancer with a suspicious phenotype, to detect this rare condition [10].

While the correlation between BRCA mutations and Fanconi anemia has been documented in literature, the association with the development of hematological malignancies has been debated over the last 10 years and is still a topic of discussion. BRCA1/2 mutations prevalence in the female population has been estimated at around 1 in 300 to 500 women [11], 0.32 % estimated carriers for BRCA1, 0.69 % for BRCA2 [12]. These mutations account for 5–10 % of all breast cancer cases and up to 15 % of all ovarian cancer cases [13], and estimated cumulative lifetime incidence of breast and ovarian malignancies are respectively 90 and 24 % for BRCA1-mutations carriers, and 41 and 8.4 % for BRCA2-mutations carriers [12]. Framed the problem, in the 2019 updated recommendation statement, the US Preventive Services Task Force assessed that should receive genetic counseling and test for BRCA mutations all women with a familiar history of BRCA mutations and/or personal or familiar history of not only breast and ovarian but also tubal or peritoneal cancer [11]. Indeed, in the last few years, attention has been focused on cancers other than breast and ovarian, and the data concerning hematological malignancies still remain discordant.

In 2005 Friedenson B. made the first effort to systematize the available data on the risk of developing “all cancers except breast or ovary” in these subjects [14]. Focusing on hematological oncology, four main studies were taken into consideration. The first in 1994, when Goldgar D.E. et al. assessed a relative risk (RR) of 1.92 of developing non-Hodgkin lymphomas (NHL) in 1145 first degree relatives of women who had breast cancer that developed before 50 years [15]. In 2000 Shih H. et al. found an association between BRCA1/2 mutations and leukemia, with 5 cases out of 98 with breast cancer reporting at least 1 other primary cancer in themselves or a relative with breast cancer, 2 of which were BRCA1/2 mutated [16]. In the other two studies, both dated 2001, the authors calculated that BRCA1- and BRCA2-mutated patients have respectively an RR of 2.31 and 2.6 of developing acute myeloid leukemia (AML) [17, 18]. Risch et al. in 2006 estimated the RR to be 3.7 in BRCA1-mutated patients [12].

Analyzing retrospectively different cohorts of patients, many authors through the years gave evidence of the fact that damage in each step of the BRCA pathway is associated with and can lead to the development of hematological malignancies [19, 20]. The study of family history for breast and ovarian cancer has placed the familiarity itself in the array of risk factors for AML [15, 21, 22]. From another point of view, BRCA pathway defects were found in leukemogenic cells, as proof of its impairment in leukemic transformation [23]. In particular, a reduced BRCA1 expression was documented in primary and secondary AML, and, more recently, an increased miR-155 expression, an oncogenic miRNA, which may have an additional role in leukemogenesis [20, 24, 25]. Moreover, the study of BRCA2, along with other FA genes, in therapy-related acute myeloid neoplasms showed that heterozygous carriers of FA variants may have increased susceptibility to environmental carcinogens and to the DNA-damaging action of cytotoxic therapy used to treat primary tumors, possibly leading to leukemogenesis [26]. Finally, the investigation of cohorts of BRCA-mutated subject highlighted in most cases the presence of a possible causal link between BRCA functions impairment and leukemia/lymphoma [12, 16–18, 27, 28].

Nevertheless, some studies did not confirm those data, keeping the debate open on whether healthy BRCA-mutated subjects should be considered at higher risk of developing hematological malignancies. In 2002 Thompson et al. analyzed a cohort of 11,847 individuals from 699 families segregating a BRCA1 mutation across Europe and North America, finding no significative association with hematologic disorders, indeed showing a decreased incidence of NHL in carriers compared to noncarriers (RR 0.23 (0.09 to 0.6); *p* value < 0.001) [29].

In 2016 an International Prospective Cohort Study was conducted to assess the incidence of leukemia in women with BRCA1/2 mutations. They evaluated 7243 women with a BRCA1 or a BRCA2 mutation, coming across five cases of leukemia (two BRCA1, three BRCA2), with the actual risk of developing it < 1 %. Compared to the general population, the risk of leukemia was fivefold higher for women who had prior BRCA2-associated breast cancer not treated with chemotherapy (SIR = 4.76 (1.21–12.96) *P* = 0.03), and eight-fold higher for women who received chemotherapy for BRCA2-associated breast cancer (SIR = 8.11 (2.06–22.07) *P* = 0.007). For BRCA1 mutation carriers, the risk of leukemia was similar to the general population. One possible explanation could be found in the genomic instability resulting from BRCA pathway disruption, which compromises the proliferation ability of leukemic cells [30].

### Hematopoietic stem cells and their reconstitution

Patients with BRCA1 and BRCA2 haploinsufficiency, looking at the role that these genes have in DNA repair, have been considered more likely to have longer and deeper hematologic toxicity after chemotherapy. Analyzing the problem from a clinical point of view, several small studies have reported conflicting data as to whether women with a single inherited mutation in BRCA1 or BRCA2 experience excess hematologic toxicity during cytotoxic chemotherapy [31–34]. In almost all the studies neutropenia after treatment was detected more frequently in BRCA-mutated patients than in controls, but conclusions were limited by small sample size, lack of a comparable control population, and/or inability to evaluate those with BRCA1 vs. BRCA2 mutations separately. Recently, wider cohorts have been analyzed to shed light on this problem.

Two retrospective studies compared BRCA mutation carriers and noncarriers treated with platinum-based chemotherapy. The first one showed that BRCA mutation carriers were more likely to experience an absolute neutrophil count below 1.0 × 10/L than noncarriers (*P* = 0.02) [35], but no differences in hematological toxicities. In the second one the authors observed a significantly higher frequency of pancytopenia in BRCA mutated patients than in controls, and needed a higher percentage of granulocyte-colony stimulating growth factors (G-CSF) injection and dose delay [36].

Finally, a multicenter retrospective matched cohort study detected similar frequency, severity, and timing of hematologic toxicities during curative intent breast cancer chemotherapy in the matched groups, suggesting that BRCA1 or BRCA2 haploinsufficiency is adequate to reconstitute hematopoiesis in the face of short-term repetitive hematopoietic stressors [37].

While haploinsufficiency could be investigated from a clinical point of view, the effects of complete BRCA1 deficiency on bone marrow (BM) function in humans are still unknown. Over the last few years, to explore the ability of BRCA-mutated subjects to reconstitute hematopoiesis of lethally-irradiated recipients, different murine models have been created.

In 2013 Bai L. et al. showed in BRCA transgenic mice that BRCA1 downregulates the expression of cycle cell inhibitors of the Cip/Kip family, which can restrain the entry of HSCs entry into S phase and maintain the quiescence of HSCs. In this study, the competitive transplantation assay demonstrated that BRCA1 donor-derived HSCs failed to reconstitute the hematopoiesis in the recipients. Finally, mutation of BRCA1 induced a decrement in the percentage of B cells and lymphoid progenitors [38].

In 2016 Vasanthakumar A. et al. created different breeds of BRCA1 mutated mice, homozygous, haploinsufficient, and normal unmutated controls, trying to detect differences in the ability to induce hematopoiesis between the three groups. They showed a reduced capacity for the BRCA^−/−^ bone marrow cells to form hematopoietic colonies in vitro and to reconstitute hematopoiesis in irradiated recipients, consistent with a hematopoietic progenitor functional defect; normal hematopoiesis reconstitution was seen in BRCA1^+/+^ and BRCA1^+/−^ mice [39]. Nevertheless, they didn’t test whether BRCA1 is required for HSC function or whether heterozygosity for BRCA1 mutations affects recovery after chemotherapy in humans or mice.

With this background, in 2017, Mgbemena V.E. et al. tested the impact of different BRCA mutations on HSC function and whether heterozygosity for BRCA1 mutations affects recovery after chemotherapy in humans or in mice. They found that homozygous mice had a significant reduction in bone marrow cellularity compared to controls, with a slight but significant reduction of white blood cell (WBC) and lymphocyte levels also in haploinsufficient mice; HSC frequency was not increased in the spleens either, so this decline in HSC frequency did not reflect HSC mobilization, and the frequency and the absolute number of hematopoietic progenitor cells was also severely reduced. To test the sensitivity to DNA stress, they treated transgenic mice with chemotherapy looking at abnormalities in the recovery and tested the capacity to reconstitute irradiated mice. Fully mutated cells were not able to reconstitute hematopoiesis, with no detectable donor-derived cells in the peripheral blood after 8 weeks. No significant differences were reported between haploinsufficient and control cells in the reconstitution of primary recipient mice, but after serial bone marrow transplantation was observed a deleterious effect of proliferative stress on Brca1 haploinsufficient HSCs. Finally, developing a humanized Brca1 allele, they found that the BRCA15382insC protein, derived from a common Ashkenazi Jewish founder mutation of the BRCA1 gene, is more deleterious to hematopoietic stem and progenitor cells than the null allele mutation [40]. HSCs from mice with mutations in DNA damage repair proteins that also lead to cancer susceptibility syndromes, such as Brca2 [41], have defects in their ability to reconstitute bone marrow in irradiated mice, and mice with mutant Rad50 exhibit hematopoietic failure [42]. However, the hematopoietic phenotype they observed after Brca1 deletion was much more severe than the phenotypes reported in these studies.

## Discussion

Could haploinsufficient BRCA mutations have a “hematological impact” in humans? Are BRCA-mutated donor HSCs sufficient to reconstitute hematopoiesis in conditioned recipients? The evidence gathered over the years fail to give a clear answer to these questions; indeed, they leave open to discussion indications that any hematologist might face in clinical practice. Nonetheless, based on the available data, some considerations can be made.

The role of the BRCA mutations in the Fanconi anemia has been studied and elucidated through the years. Even if the molecular pathway was very suggestive for a correlation between mutational status and the possible onset of the disease, only the actual clinical observation led, a few years later, to the individuation of a specific hematological disease in patients with both alleles mutated [9]. The mutation carriers, indeed, did not show any sign of the typical phenotype of the FA. As well as for FA, the healthy carriers of the BRCA mutations don’t seem to be at higher risk of developing hematological malignancies. The situation is different for oncologic BRCA-mutated patients, treated with chemotherapy or not, for whom a risk of developing secondary leukemia/lymphoma is significant, in particular for BRCA2-mutated patients [30]. These data suggest that BRCA mutational status can be considered as a risk factor for hematological malignancies, but only as secondary malignancies and/or in the presence of bone marrow stress factors.

Great efforts have been made to understand whether haploinsufficient patients undergo deeper and longer hematologic toxicity than the unmutated counterparts. Most of the studies found a higher incidence of neutropenia in carriers, often associated with anemia and thrombocytopenia, which occasionally required G-CSF, but the only matched study, as well as the largest, showed no differences between carriers and noncarriers [37]. The heterogeneity of the treatments, the retrospective nature of the studies and the exiguity of the cohorts made it difficult to reach a consensus on this specific topic, although on a clinical basis we could say, with all the limitations of the casuistry, that the reconstitution of hematopoiesis is not compromised in carriers facing short-term bone marrow stress.

The study of murine models knocked for BRCA1/2 allowed us to have a deeper view of how these mutations affect HSC frequency and function. On the one hand, studies showed that fully mutated BRCA mice not only fail to reconstitute lethally irradiated recipients, but they also have lower WBC, absolute neutrophils count and platelets than controls. On the other hand, the heterozygosity for a null allele didn’t affect reconstitution, showing results superimposable with controls after transplantation. It is interesting to note that serial transplantations, implying a higher stress factor for HSC, had a deleterious effect on haploinsufficient HSC and that mice knocked for a human BRCA mutation showed an HSC phenotype worse than the null allele counterparts [40].

There are several limitations of this review: the retrospective nature of the studies, the exiguity of the cohorts, the heterogeneity of the treatments and finally the limitation related to the murine models. Considering all these limitations, these acquisitions still allow some considerations to be made. In the clinical practice, we know that a short time bone-marrow stress doesn’t compromise reconstitution, but we don’t know if a conditioning regimen could be compensated by BRCA mutated HSCs, even if murine models showed no differences between haploinsufficient and normal HSCs. This consideration is even more legitimate in the light of the fact that different mutations correspond to different phenotypes, sometimes worse than null alleles (as we have seen in Ashkenazi’s population).

Transposing all these assessments to clinical practice inevitably raises ethical concerns that clinicians have to take into account when approaching this clinical issue: What is the suggested algorithm of the donor choice? Should a matched unrelated donor be preferred? Is a mismatch allowed if a healthy BRCA1/2 mutation carrier related donor is available? Should the potential recipient be enlightened about the problem, have a choice, and give his consent? Will a healthy be a safe donor to a BRCA1/2 positive recipient?

Based on the literature data, it could be questionable to use the HSCs of a BRCA-mutated donor in the presence of another available donor with the same compatibility, given the downregulating effect of the mutations on hematopoiesis. Surely the patient should be informed of the problem and give his consent: all the considerations made constitute the basis for arriving at a first conclusion of *clinical equipoise* or of a reasonable clinical uncertainty within the scientific community regarding which is the best choice for the patient; this therefore places the ethical decision-making criterion of “medical indications” at least on the same level as the “patient’s preferences” who becomes the appropriate decision maker within an interdisciplinary clinical ethics consultation. Nevertheless, if the recipient suffers from a disease with an otherwise rapidly fatal outcome in the absence of any other donor or otherwise having to choose a donor with inferior compatibility, it may be necessary to opt for *quoad vitam* treatment rather than postpone it due to BRCA mutations.

## Conclusions

There is no strong evidence to firmly support or discourage the hematopoietic stem cell donation, independently from source (i.e. bone marrow, peripheral blood or cord blood units) by BRCA1/2-mutation carriers. Our attitude is to look for an alternative donor in patients carrying these mutations, mostly as precautionary approach. The choice of whether or not to carry out a haploidentical transplantation from a BCRA-wildtype donor, should take into consideration all the clinical and ethical aspects of the issue, and, if possible, it should include a multidisciplinary evaluation with experts on marrow transplantation and medical ethics. One additional and final issue that remains unanswered is whether or not these donors if candidates should donate bone marrow or G-CSF mobilized peripheral blood stem cells. Collecting more data retrospectively and then designing a prospective trial to demonstrate whether BRCA-mutated carriers’ HSCs are inferior or not, would be the only way to finally shade light upon this issue.

## Data Availability

Not applicable.
